# Socio-demographic factors associated with underweight and stunting among adolescents in Ethiopia

**DOI:** 10.11604/pamj.2015.20.252.3588

**Published:** 2015-03-17

**Authors:** Huruy Assefa, Tefera Belachew, Legesse Negash

**Affiliations:** 1 Department of Public Health, College of Health Sciences, Mekelle University, PO. Box, Mekelle, Ethiopia; 2 Department of Population and Family Health, College of Public Health and Medical Sciences, Jimma University, PO. Box 1104, Jimma, Ethiopia; 3 Department of Statistics, College of Natural Sciences, Jimma University, PO.Box 1104, Jimma, Ethiopia

**Keywords:** Adolescent, nutritional status, linear regression, Jimma, Southwest Ethiopia

## Abstract

**Introduction:**

Nutrition during adolescence plays an important role in the individual's life. There are different factors that affect nutritional status of adolescents. Socio Economic Status, age, sex and mothers’ educational level are among the important determinants factors of nutritional status of adolescents. Younger adolescents tend to be more undernourished than older adolescents, and, contrary to expectations that boys are almost twice as undernourished as girls. In this study, we test the competing hypothesis about the correlates of nutritional status among Ethiopian adolescents.

**Methods:**

We report a total of 2084 adolescents from the second round of a 5-year longitudinal study in Jimma zone, southwest Ethiopia. Univariate and Multivariable linear regression were used to assess socio-demographic factors associated with Underweight and Stunting among Adolescents in Jimma zone.

**Results:**

Age, highest grade completed, job and last attended in community school were positively associated with BMI for Age z-score and highest grade completed, household income and job were positively associated with Height for Age z-score. However, male gender was negatively associated with BMI for Age z-score and male gender, last attended in community school, abdominal pain and household size were negatively associated with Height for Age z-score.

**Conclusion:**

Age of the adolescents, gender, educational status, employment status and type of last school attended are associated with underweight; and gender, place of residence, household size, household income, educational status, employment status, type of last school attended and abdominal pain are associated with stunted. The findings reflect socio-demographic characteristics are associated with underweight and stunting.

## Introduction

The world population is believed to have reached over 6.6 billion [1]. Adolescence is a particularly unique period in life because it is a time of intense physical, psychosocial, and cognitive development. Nutrition during adolescence plays an important role in the individual's life. Increased nutritional needs to adolescents gain up to 50% of their adult weight, more than 20% of their adult height, and 50% of their adult skeletal mass [2].

There are different factors that affect nutritional status of adolescents. Socio Economic Status, age, sex and mothers’ educational level are among the important determinants factors of nutritional status of adolescents [3–6]. Studies have shown that adolescent women from low economic status households were most affected by malnutrition and the higher the level of education, the lower the proportion of undernourished adolescents women and rural adolescent women are more likely to suffer from chronic energy deficiency than adolescents women in urban areas [7, 8].

There are only a handful of studies on adolescent malnutrition in developing countries- there is indicates that younger adolescents tend to be more undernourished than older adolescents, and, contrary to expectations that boys are almost twice as undernourished as girls. In addition, these few studies suggest that there are more undernourished adolescents in Sub-Saharan Africa, and a higher prevalence in rural than in urban areas [9].

In Ethiopia, undernourishment among preschool aged children has been well documented [10], but studies on factors affecting the nutritional status of adolescents have not been studied in detail [11]. The available studies focused on factors affecting pregnancy outcomes rather than on problems associated with normal growth and development of adolescents [12, 13]. A recent study on the nutritional status of adolescent girls from rural communities of Tigray, Northern Ethiopia [5] has helped to close the research gap in the area, although it is still focused only on rural adolescent girls, on small geographic area and small populations. This study is expected to play important role in identifying the socio-demographic factors associated with Underweight and Stunting among Adolescents of Jimma zone, south west Ethiopia. In this study, we test the competing hypothesis about the socio-demographic factors associated with Underweight and Stunting among Ethiopian adolescents.

## Methods

### Study sample

A community-based cross-sectional study was conducted from 2010 adolescents enrolled in the second round of the five year longitudinal study of adolescents in Jimma zone, Southwest Ethiopia. A census was done to generate the list of all households which gave a sampling frame for random selection of 3,700 households from the total of 5,795 households in the list. A two-stage sampling plan was used to select the target sample of adolescents. Households were classified into urban (Jimma City), semi-urban (Serbo, Dedo and Yebbu Towns) and six rural communities in the vicinity of the small towns. At the first stage, households were randomly sampled with the sample size in each kebele determined by the relative proportion of the study population in the kebele and the overall target sample size. In the second stage, one adolescent (a boy or a girl) was randomly selected from each household using a Kish Table [14]. Using this sampling strategy a total of 1059 boys and 1025 girls were interviewed in round one. This paper reports on all the 2084 adolescents in the second round of the five year longitudinal family survey of youth.

### Measurements

Structured adolescent level questionnaires were used to collect data. The questionnaires were interviewer-administered and translated in to Amharic and Oromifa languages and checked for consistency by another person who speaks both Oromifa and English. The questionnaire focused on issues related to adolescents’ experiences of Nutritional status, socio-demographic, health, food insecurity and anthropometric measurements of the adolescent. The interview was conducted in a private place by an interviewer of the same sex.

The interviewers received one week of intensive training prior to the pretest and an additional week of training was given with the final version of the questionnaire before the start of the actual interviews. Supervisors checked the data collection process and filled questionnaires daily to ensure accuracy of the data. The research team supervised the data collection team every week through meetings and checking of the filled questionnaires.

We measured height to the nearest 0.1 cm using a stadiometer (SECA, Hannover Germany) and weight to the nearest 0.1 kg using digital scales (SECA). The level of stunting (height for age z-scores), which is an indicator of chronic malnutrition and wasting (weight for age z-scores), which is another indicator of malnutrition were calculated using WHO Athro-Plus software [15]. Thus, those below -2 standard deviations of the NCHS median reference for height-for-age and weight-for-height were defined as stunting and wasting, respectively.

Socio-demographic variables like age, sex, Place of residence, marital status, whether the adolescents have a child, highest grade completed, type of school completed, religion, whether the adolescents have a job, household income, household size, mother's education status and father's education status), health related variables like Fever, Cough, Fast Breathing, Diarrhea, Vomiting, unable to drink or eat, Ulcer, Depression, sleep under insecticide treated mosquito bed net, currently smoke tobacco, does any member of the household smoker and food security variables were recorded. The questionnaire was tested on 200 adolescents (not included in the sample) selected from a community in Jimma city.

### Data analysis

The data were double entered, checked for missing values and outliers using SPSS (SPSS Inc. version 16.1, Chicago, Illinois); and analyzed using SAS version 9.2. First, univariate linear regression analyses were conducted. To identify the predictors of Underweight (BMI for Age z-score) and Stunting (Height for Age z-score), multivariable linear regression analysis with stepwise variable selection and AIC criteria procedure was employed and variables that showed a statistically significant (P < 0.05) in the univariate analyses were entered to the multivariable linear regression.

Goodness of fit of the models was assessed using Akaike's information criterion (AIC) and Adjusted-R^2^ and Partial regression residual plots showed that all had linear relationship. Normality of the data was assessed using a Q-Q plot and there was no need of transformation; and Outliers and Influential observations were not as such influential and then retained in the final model. Co-linearity between predictor variables were checked using variance inflation factor (VIF); and Interaction and confounding variables were checked. All tests were two-sided and a P < 0.05 was considered statistically significant. We present the results of the linear regression as parameter estimates (ß), P-values and 95% confidence intervals.

### Ethical consideration

Informed verbal consent was obtained both from the parents and each adolescent before the interview or measurement. The study obtained ethical clearance from the Ethical Review Board of Jimma University (Ethiopia). The head of the household was interviewed by using the household questionnaire.

We were not obtained written verbal consent, because most of the respondents did not write and read well. We first read the consent paper and explain the objective of the study in front of each respondent and after they agree on the objective of the study and they signed. The ethics committees also approved the consent procedure after we explain the procedures.

## Results

Of 2084 adolescents included in the study (Table 1), 1951 and 1956 adolescents’ complete data were available for BMI for Age z-score and Height for Age z-score. Out of 1951 and 1956 adolescents covered in the study for BMI for Age z-score and Height for Age z-score 1577 (80.83%) and 313 (16%) were found to be underweight and stunted respectively.

**Table 1:** Socio demographic characteristics of nutritional status of adolescents, Jimma zone, South West Ethiopia, 2011

		Prevalence (%)			Prevalence (%)		
Variables	Category	Underweight (HAZ < -2)	Total	P-value	Stunting (HAZ < -2)	Total number of Adolescents	P-value
place of Residence	urban	620 (83)	743		88 (12)	743	
	Semi-urban	459 (84)	545		90 (16)	546	
	Rural	498 (75)	663	p < 0.001	135 (20)	667	p < 0.001
Marital Status	single	1571 (81)	1945		313 (16)	1950	
	married	6 (100)	6	0.232	0 (0)	6	0.284
Sex	Female	839 (89)	941		103 (11)	942	
	Male	738 (73)	1010	p < 0.001	210 (21)	1014	p < 0.001
Ever had child	No	1568 (81)	1941		312 (16)	1945	
	Yes	9 (90)	10	0.460	1 (9)	11	0.794
Type of last school attended	Government	1511 (81)	1866		291 (16)	1870	
	Private	16 (94)	17		4 (24)	17	
	Community	4 (100)	4		0 (0)	4	
	other	46 (72)	64	0.680	18 (28)	65	0.003
Religion	Muslim	918 (79)	1165		191 (16)	1170	
	Orthodox	562 (85)	664		104 (16)	664	
	Protestant	91 (81)	113		16 (14)	113	
	other	6 (67)	9	0.008	2 (22)	9	0.681
Job	no	1117 (80)	1398		234 (17)	1399	
	Yes	460 (83)	553	0.097	79 (14)	557	0.166
Fathers’ education	no education	487 (80)	611		188 (17)	1089	
	primary	633 (79)	800		102 (16)	624	
	secondary	371 (84)	441	0.086	19 (9)	213	0.018
Mothers’ education	no education	860 (79)	1086		112 (18)	614	
	primary	513 (82)	623		137 (17)	801	
	secondary	178 (84)	212	0.127	53 (12)	441	0.010
Age in years (± SD)		14.78(±1.34)	2083	0.001	14.78(±1.34)	2083	p < 0.001
Highest grade completed (± SD)		5.16(± 2.66)	2084	p < 0.001	5.16(± 2.66)	2084	p < 0.001
Household income (± SD)		105.77(± 188.13)	2084	0.686	105.77(± 188.13)	2084	0.539
Household size (± SD)		8.49(± 3.42)	2084	0.021	8.49(± 3.42)	2084	0.166

Most of the underweight adolescents (Table 1) were females (53.20%), reside in urban (39.32%), single (99.68%), Muslim by religion (58.21%), no child (99.43%), attended last school in government (95.82%), no job (70.83%), adolescent father‘s education had primary (42.45%) and adolescent mother‘s education had no education (55.45%). And most of the stunted adolescents (Table 1) were males (67%), reside in rural (43%), single (100%), Muslim by religion (61%), no child (99.68%), attended last school in government (93%), no job (75%), adolescent father‘s education had no education (61%) and adolescent mother‘s education had primary (45%).

The mean (+ SD) age of the adolescents (Table 1) were 14.78(+1.34) and the mean (+ SD) highest grade completed, household income, and household size of the adolescents were 5.16(+ 2.66), 105.77(+ 188.13) and 8.49(+ 3.42) respectively for both underweight and stunted adolescents. Of the underweight adolescents (Table 2), fever (86%), cough (43%), vomiting (35%), unable to eat or drink (49%) and abdominal pain (31%) were the health problems in the study area. And of the 313 stunted adolescents, fever (85%), cough (49%), vomiting (35%), unable to eat or drink (45%) and abdominal pain (39%) were the health problems. Only 21% of the underweight and stunted adolescents did not secure their food in the study area.

**Table 2:** Health related and food insecurity characteristics of nutritional status of adolescents, Jimma zone, South West Ethiopia, 2011

		Prevalence (%)			Prevalence (%)		
variables	Category	Underweight (HAZ < -2)	Total	p-value	Stunting (HAZ < -2)	Total	p-value
Diarrhea	no	1372 (87)	1696		269 (86)	1701	
	yes	205 (13)	255	0.849	44 (14)	255	0.558
Fever	no	228 (14)	284		48 (15)	286	
	yes	1349 (86)	1667	0.799	265 (85)	1670	0.697
Cough	no	903 (57)	1101		161 (51)	1105	
	yes	674 (43)	850	0.130	152 (49)	851	0.049
Fast breathing	no	1191 (76)	1454		239 (76)	1459	
	yes	386 (24)	497	0.038	74 (24)	497	0.433
Vomiting	no	1022 (65)	1259		204 (65)	1264	
	yes	555 (35)	692	0.601	109 (35)	692	0.823
Could not eat or drink	no	804 (51)	965		159 (51)	970	
	yes	773 (49)	986	0.006	154 (49)	986	0.641
Abdominal pain	no	1086 (69)	1327		192 (61)	1332	
	yes	491 (31)	624	0.099	121 (39)	624	0.005
Genital discharge or ulcer?	no	1562 (99)	1931		310 (99)	1936	
	yes	15 (1)	20	0.506	3 (1)	20	0.902
Depression/extreme sadness/worry?	no	1232 (78)	1529		242 (77)	1534	
	yes	345 (22)	422	0.586	71 (23)	422	0.603
Sleeps under insecticide treated bed net	no	1376 (87)	1709		282 (90)	1714	
	yes	201 (13)	242	0.347	31 (10)	242	0.148
Smoking	no	1571 (99.62)	1941		311 (99)	1946	
	yes	6 (0.38)	10	0.093	2 (1)	10	0.730
Anyone in household smoke tobacco products	no	1377 (87)	1707		276 (88)	1712	
	Yes	200 (13)	244	0.630	37 (12)	244	0.703
Adolescent food insecurity	secure	1247 (79)	1549		247 (79)	1554	
	non-secure	330 (21)	402	0.472	66 (21)	402	0.799

In bivariate linear regression models (Table 3), residence in semi-rural, age, highest grade completed, last attended in community school, household income, adolescent mothers with secondary education and adolescent fathers with secondary education were positively associated with BMI for Age z-score, while residence in rural, male gender and Household size were negatively associated with BMI for Age z-score.

**Table 3:** Predictors of BMI for age z-scores in adolescents in Jimma zone, South West Jimma

	Univariate Linear Regression				Multivariable Linear Regression			
Variables	ß	95% CI		P-value	ß	95% CI		P-value
Residence in semi urban	0.1283	0.0124	0.2443	0.0301	0.0820	-0.0492	0.2132	0.2204
Residence in rural	-0.3132	-0.4223	-0.2041	0.0000	-0.0990	-0.2628	0.0649	0.2363
Sex (male)	-0.7330	-0.8321	-0.6340	0.0000	-0.7419	-0.8434	-0.6404	0.0000
Age in Years	0.1131	0.0746	0.1517	0.0000	0.0594	0.0173	0.1015	0.0057
Highest grade completed	0.0685	0.0489	0.0881	0.0000	0.0554	0.0290	0.0817	0.0000
private school	0.0278	-0.4102	0.4658	0.9009	0.1677	-0.2662	0.6016	0.4485
community school	0.7070	0.0119	1.4020	0.0462	0.7912	0.0238	1.5585	0.0433
Other type of school	-0.4743	-1.0516	0.1029	0.1072	0.2767	-0.2914	0.8447	0.3396
Household income	0.0004	0.0001	0.0006	0.0072	0.0001	-0.0002	0.0004	0.6156
Job	0.1144	-0.0011	0.2298	0.0523	0.2121	0.0967	0.3275	0.0003
Abdominal pain	-0.0766	-0.1883	0.0350	0.1783	0.0073	-0.1006	0.1151	0.8950
household size	-0.0233	-0.0384	-0.0081	0.0026	-0.0091	-0.0253	0.0070	0.2662
Mothers with primary education	0.0120	-0.1001	0.1240	0.8344	-0.0452	-0.1717	0.0813	0.4838
Mothers with secondary education	0.1706	0.0033	0.3379	0.0456	-0.0270	-0.2273	0.1733	0.7914
Fathers with no education	-0.0702	-0.1837	0.0433	0.2254	0.1205	-0.0103	0.2513	0.0710
Fathers with secondary education	0.1743	0.0492	0.2995	0.0063	0.0312	-0.1144	0.1767	0.6745

Source: Jimma Longitudinal Family of Youth; Round 2, 2006-2007 CI= confidence interval

After adjusting for all other variables in the multivariable linear regression model (Table 3), age, highest grade completed, job and last attended in community school were positively associated with BMI for Age z-score. However, male gender was negatively associated with BMI for Age z-score. The effect of residence in semi-urban, residence in rural, household income, household size, adolescent mothers with secondary education and adolescent fathers with secondary education disappeared in the multivariable linear regression model.

### Determinants of nutrition status of adolescents in terms of height for age z-scores

In bivariate linear regression models (Table 4), age, highest grade completed, household income, job and adolescent fathers with secondary education were positively associated with BMI for Age z-score, while residence in rural, male gender, last school attended in community school, abdominal pain and household size were negatively associated with the Height for Age z-score.

**Table 4:** Determinants of nutrition status of adolescents in terms of height for age z-scores in Jimma zone, South West Ethiopia

	Univariate Linear Regression				Multivariable Linear Regression			
Variables	ß	95% CI		P-value	ß	95% CI		P-value
Residence in semi urban	0.0041	-0.1065	0.1148	0.9417	-0.0487	-0.1794	0.0819	0.4645
Residence in rural	-0.2136	-0.3178	-0.1093	0.0001	0.1136	-0.0495	0.2768	0.1721
Sex (male)	-0.1473	-0.2464	-0.0482	0.0036	-0.1595	-0.2606	-0.0585	0.0020
Age in Years	0.0638	0.0268	0.1007	0.0007	-0.0320	-0.0739	0.0100	0.1350
Highest grade completed	0.0917	0.0732	0.1101	0.0000	0.1066	0.0803	0.1328	0.0000
private school	-0.3180	-0.7355	0.0996	0.1355	-0.1189	-0.5510	0.3133	0.5897
community school	-0.8765	-1.5391	-0.2140	0.0095	-1.2114	-1.9757	-0.4471	0.0019
Other type of school	-0.5280	-1.0622	0.0062	0.0527	-0.0004	-0.5486	0.5478	0.9988
Household income	0.0006	0.0003	0.0008	0.0000	0.0005	0.0002	0.0007	0.0014
Job	0.1258	0.0159	0.2356	0.0248	0.2198	0.1051	0.3345	0.0002
Abdominal pain	-0.1451	-0.2514	-0.0388	0.0075	-0.1328	-0.2401	-0.0255	0.0153
household size	-0.0254	-0.0398	-0.0110	0.0006	-0.0224	-0.0384	-0.0064	0.0062
Mothers primary education	-0.0649	-0.1720	0.0422	0.2347	-0.0963	-0.2222	0.0296	0.1336
Mothers secondary education	0.2982	0.1389	0.4574	0.0002	0.0747	-0.1248	0.2741	0.4628
Fathers no education	-0.0874	-0.1959	0.0211	0.1144	0.0716	-0.0585	0.2018	0.2805
Fathers secondary education	0.1536	0.0337	0.2735	0.0121	-0.0804	-0.2253	0.0646	0.2770

Source: Jimma Longitudinal Family of Youth; Round 2, 2006-2007. CI= confidence interval

After adjusting for all other variables in the multivariable linear regression model (Table 4), highest grade completed, household income and job were positively associated with Height for Age z-score, while male gender, last attended in community school, abdominal pain and household size were negatively associated with Height for Age z-score. The effect of adolescents’ residence in rural, age of the adolescent, adolescent mothers with secondary education and adolescents fathers with secondary education disappeared in the multivariable linear regression model.

## Discussion

Our results showed that, age was positively associated with BMI for Age z-score. An association of age with BMI for Age z-score has previously been reported [5, 8]. The risk of underweight was, on average, significantly higher for younger adolescents than older adolescents.

Studies have suggested a positive association between BMI for Age z-score and educational status and employment [7, 8, 12, 16]. Our study found that highest grade completed was positively associated with BMI for Age z-score and Height for Age z-score. They indicated that adolescents who receive even a minimal education are generally more aware than those who have no education of how to utilize available resources for the improvement of their own nutritional status and that of their families. Education may enable to make independent decisions, to be accepted by other household members, and to have greater access to household resources that are important to nutritional status [16].

Our study found that job was positively associated with BMI for Age z-score and Height for Age z-score. The risk of underweight and stunted was significantly high, on average, for unemployed adolescents than employed adolescents in Jimma zone. This finding is consistent with other studies [17]. They indicated that unemployment is a significant factor for chronic energy deficiency in these adolescents as compared with employed adolescents. Our study also found that adolescents attended their last school in community school was positively associated with BMI for Age z-score. Studies have demonstrated the connection between Height for Age z-score and household income [6–8]. Our study also indicated Household income was positively associated with Height for Age z-score.

We found male gender was negatively associated with BMI for Age z-score and Height for Age z-score, as previously reported by others [4]. The risk of underweight was, on average, significantly higher for adolescent males than adolescent females. This may be because of the fact that biological, behavioral, and socio-cultural mechanisms have been proposed for the gender differences in morbidity and mortality. Biologically, female subjects have an advantage for better health and longer survival because of the role of sex hormones in modulating lipid levels and increasing immune response. In addition, the difference in morbidity and mortality between boys and girls is further related to individual lifestyle, the use of health care, and health and illness behaviors and practices. For example, adolescent boys are more likely to smoke and have higher propensities of taking greater risks that expose them to injury.

The result also showed that residence in rural was negatively associated with the Height for Age z-score which is consistent with reports of studies in Ethiopia and Sub-Saharan Africa [8, 9, 17, 18]. The observed urban-rural difference could be an indication of low access and use of health services in the rural areas as compared with urban areas. In general, people living in cities have better health and lower death rates than rural residents, even though the urban poor often live in unsanitary and crowded conditions. Compared with rural residents, urban residents have better access to medical services and are more easily reached by immunization and educational campaign.

We found also household size was negatively associated with the Height for Age z-score, as previously reported by others [3, 6]. In addition, abdominal pain and last school attended in community were negatively associated with the Height for Age z-score.

We acknowledge a number of limitations in our study. As the study involved adolescents who are at the different stages of academic status, we used the highest grade completed as a measure of educational attainment that can serve across all age groups. However, there are other measures of educational attainment that were not captured. The fact that we did not have data from the school records regarding the academic performances is also a limitation of our analysis.

## Conclusion

In conclusion, the study revealed age of the adolescents, gender, educational status, employment status and type of last school attended were associated with underweight; and gender, place of residence, household size, household income, educational status, employment status, type of last school attended and abdominal pain were associated with stunted. Thus, underweight and stunting is the reflection of socioeconomic development demanding combination of different types of policies and programs for its solution. To reduce and prevent underweight and stunting Strategies need to involve and more targeted interventions. Promoting gender equality through effective behavior change communications needs to be considered. Further research will help to understand to identify the most effective strategies for reducing adolescent malnutrition in the study area.
